# Prevalence and factors associated with dyslipidemia in patients with first hospitalization for major depressive disorder: a large sample cross-sectional study

**DOI:** 10.1186/s12888-024-05848-3

**Published:** 2024-05-27

**Authors:** Huimin Yin, Baili Lu, Kuan Zeng, Yi Li, Jun Ma

**Affiliations:** 1 Department of Psychiatry, Wuhan Mental Health Center, No. 89, Gongnongbing Road, Wuhan, Hubei Province China; 2 Department of Psychiatry, Renmin Hospital of Wuhan University, No. 99, Zhangzhidong Road, Wuhan, Hubei Province China

**Keywords:** Major depressive disorder, Hyperlipidemia, First hospitalization

## Abstract

**Background:**

Major depressive disorder (MDD) is a severe mental illness with high relapse rates and high mortality. Depression not only severely limits psychosocial functioning but also reduces quality of life. It can also negatively affect patients’ clinical parameters, including lipid metabolism markers. This study aimed to investigate the prevalence and risk factors of hyperlipidemia (HL) in patients with MDD who were hospitalized for the first time.

**Methods:**

In this study, we enrolled 981 patients with MDD who were hospitalized for the first time, collected their demographic data and biochemical indicators, and evaluated their clinical symptoms. We divided the patients into HL and non-HL subgroups based on whether they had co-morbid HL. We compared whether there were significant differences between the two groups regarding demographics and general clinical information.

**Results:**

A total of 708 of 981 MDD patients were described as being in the hyperlipidemic group, with an incidence of 72.17%. Clinical Global Impression Scale-Severity of Illness (CGI-SI) score and Hamilton Depression Scale (HAMD) score are risk factors for co-morbid HL in patients with MDD. The area under the ROC curve for the CGI-SI and HAMD score and their combined discriminatory ability was approximately 63%, 67%, and 68%, respectively.

**Conclusion:**

The prevalence of HL was high in patients with MDD who were first hospitalized; Higher HAMD score and CGI-SI score were risk factors for the development of HL in MDD; The HAMD score and the CGI-SI score are predictive of the severity of HL.

## Introduction

Major depressive disorder (MDD) is a severe mental illness, the course of which is usually chronic and recurrent and has a high mortality rate [1]. It is estimated that the current prevalence of MDD in the world is 4.7%. According to a meta-analysis of the prevalence of MDD in mainland China in 2021, the point prevalence, 12-month prevalence, and lifetime prevalence of MDD in China were 1.1%, 1.6%, and 1.8%, respectively [2]. The clinical features of MDD include depressed mood, executive dysfunction, autonomic nervous system symptoms, and other symptoms, which severely limit psychosocial function and reduce the quality of life [1]. Not only that, but the study found that depression can also negatively affect the clinical parameters of the patients, especially the lipid metabolism indicators [3]. Previous studies also suggest a possible biphasic relationship between depression and hyperlipidemia (HL), with metabolic risk factors such as HL increasing the risk of depression and disorders of lipid metabolism, possibly accelerating the development of depression-like behaviors [4, 5].

The relationship between depression and abnormal lipid metabolism is complex, and the exact mechanisms are not yet precise [6]. Previous studies have shown common pathophysiological mechanisms between depression and lipid metabolism [7–10]. Oxidative stress (OS) and inflammation have become the pillars of MDD pathogenesis [8]. OS and inflammation are interdependent and complementary, and studies have shown that inflammatory cytokines are hyperactivated in MDD patients, promoting OS. At the same time, reactive oxygen species (ROS) are produced by inflammatory cells and can initiate intracellular signaling, leading to pro-inflammatory gene expression [11]. Chronic inflammation and OS have also proven to be critical factors in developing abnormalities in metabolic markers such as blood lipids [12]. Furthermore, in a study on the expression of cholesterol pathway genes and genes characteristic of atherosclerotic plaque macrophages in monocytes from MDD patients with abnormal cholesterol metabolism, the gene expression profile of monocytes of MDD patients supports a view that the low-grade inflammatory state is part of a broader immune abnormality, it is characterized by various mitochondrial dysfunctions associated with abnormal apoptosis/growth and cholesterol metabolism, as well as inflammation [13].

HL not only affects the altered physical status of MDD patients but also has a detrimental effect on the development of depression. HL is known to be a risk factor for cardiovascular disease, and data have shown that among Chinese adults, depression is associated with an increased risk of all-cause and cardiovascular disease mortality [14]. In addition, research suggests that HL may reduce treatment response to depression and predict greater depression severity [15, 16]. An animal study in rats found that hypercholesterolemia decreased acetylcholine levels, increased acetylcholinesterase activity, and increased tumor necrosis factor (TNF) and amyloid-β_42_. Behaviorally, the hypercholesterolemic rats exhibited depression-like behavior and memory loss [17]. Another animal study showed that excessive accumulation of lipids might lead to depression and anxiety-like behavior in mice by inhibiting AMPK phosphorylation and promoting a shift of mTOR toward phosphorylation to inhibit autophagy [18]. A study showed that disruption of lipids might be a potential diagnostic marker for major depression. Lipid species significantly altered were found to be increased dramatically in MDD and positively correlated with depression severity [19, 20].

So far, the occurrence of HL in MDD patients has been reported abroad. Still, the known results in existing studies deviate from the incidence of HL in Chinese MDD patients, which may be due to the different study populations and various confounding factors. Based on actual clinical practice in China, our study reported the incidence of HL among first-time hospitalized MDD patients and examined the factors influencing its occurrence.

## Materials and methods

### Subjects

Nine hundred eighty-one patients diagnosed with MDD were admitted to Wuhan Mental Health Center between July 2017 and August 2022. The inclusion criteria were: (1) Fulfill the diagnostic criteria for MDD according to the 10th revision of the International Classification of Diseases (ICD-10). (2) Have no history of hospitalization before the inpatient interview on the day of admission. (3) Be between 18 and 60 years old and of Chinese Han nationality. (4) Hamilton Depression Scale (HAMD) 17-item scores > 24 [21].

Patients were excluded from the study if they met the following conditions: (1) Pregnant or breastfeeding. (2) Having a history of substance dependence. (3) Previous or current use of hypoglycemic and lipid-lowering medications. (4)Diagnosed with severe physical diseases or personality disorders. (5) Unable to cooperate with psycho-psychological assessments due to severe behavioral disorders or other reasons.

The study received approval from the Ethics Committee of Wuhan Mental Health Center. All participants provided written informed consent, either signed by themselves or a family member.

### Research design

This study used a cross-sectional design to determine the prevalence of HL among first-time hospitalized MDD patients. Characteristics associated with HL were assessed, and demographic and general clinical data were compared between two clinical subgroups with and without HL.

We collected demographic data on the first day of patient admission for patients with MDD who met the inclusion criteria on the presentation day. These data included the patient’s age, sex, onset, duration of illness, marital status, treatment history, and education level. We collected scales capable of assessing patients’ clinical symptoms: Clinical Global Impression Scale-Severity of Illness (CGI-SI), Hamilton Anxiety Scale (HAMA), and HAMD. In addition, we collected P1-P7 of the PANSS scale. While it is true that, in general, the PANSS scale is more widely used for symptom assessment in schizophrenia. However, given that patients with MDD may experience psychotic symptoms such as self-condemnatory delusions or mood-related blame and denigration hallucinations under the influence of severe depression, we believe that it is necessary to assess P1-P7 of the PANSS scale as a basis for clinical symptom assessment.

In our study, all patients were asked to fast after 8:00 pm the previous night, and blood samples were collected from a vein. Blood pressure was measured from 6:00 to 8:00 am the following day. All blood samples were immediately sent to the hospital laboratory for testing before 11:00 am. Since the hospital laboratory performed the blood analysis, we could not obtain measurements and details of the biochemical parameters. Our biochemical indicators were limited to those collected from the medical record system. We extracted the results of the patient’s fasting biochemical parameters, specifically total cholesterol (TC), triglycerides (TG), low-density lipoprotein cholesterol (LDL-C), high-density lipoprotein cholesterol (HDL-C), fasting blood glucose (FBG) level. Since a closer correlation exists between subclinical hypothyroidism and depression [22], in patients with subclinical hypothyroidism, higher serum thyroid-stimulating hormone (TSH) also significantly modulates HDL-C, LDL-C, and TC and promotes the development of HL [23]. Therefore, we collected thyroid function indices (especially TSH, free triiodothyronine (FT_3_), and free tetraiodothyronine (FT_4_) levels) from our patients. Considering the influence of other metabolic indicators on lipids, we also collected other clinical indicators such as body mass index (BMI) [24], waist circumference (WC) [25], and blood pressure level [26] (specifically, systolic blood pressure, SBP; diastolic blood pressure, DBP).

According to 2016 Chinese guidelines for the management of dyslipidemia in adults, the thresholds for high TC and TG are 5.20 mmol/L and 1.70 mmol/L, respectively, while the thresholds for high LDL-C and low HDL-C are 3.40 mmol/L and 1.00 mmol/L. When single or multiple abnormal lipid levels are present, they are considered dyslipidemia and divided into HL and non-HL groups [27].

Relevant psychometric assessments were conducted with two uniformly trained attending psychiatrists at the medical centers where the sample was.

### Data analysis

Before performing the analysis, we checked for normality using a Quantile-Quantile Plot. We descriptively summarized the demographic and clinical characteristics of the patients and used t-tests and χ^2^ tests to compare variables between the two groups, as appropriate. In addition, we performed correlation analyses and used binary logistic regression to identify risk factors for developing HL in patients with MDD. We constructed ROC curves to assess the predictive value of the logistic regression model. Finally, we used multivariate linear regression models to identify factors affecting the severity of dyslipidemia in the target population. We used GraphPad Prism (version 8.4.3; GraphPad Software, Inc., La Jolla, CA, USA) for graphing and SPSS 26 (SPSS, Inc., Chicago, IL) for statistical analysis.

## Results

### Differences between clinical subgroups with and without HL

981 MDD patients were included in this sample, of which 708 were HL, accounting for 72.17% of the total sample. TC abnormalities accounted for 30.17% of the total; TG abnormalities accounted for 57.39%; LDL-C abnormalities accounted for 14.68%; HDL-C abnormalities accounted for 6.52%. Because two or more lipid types occur abnormally in a subset of MDD patients, the cumulative rate of subgroups exceeds the total rate of dyslipidemia. Table 1 shows the significant differences in clinical parameters between the HL and non-HL subgroups, including metabolic parameters (TSH, FBG, SBP, DBP) and significant differences between the two groups in terms of clinical symptoms (HAMA, HAMD, CGI-SI).

**Table 1:** Demographic and general clinical data of different clinical subgroups

Index	Total patients (*n* = 981)	Non-HL (*n* = 273)	HL (*n* = 708)	t/χ²	*p*
Age-years	35.62 ± 12.44	34.40 ± 11.95	36.09 ± 12.60	-1.92	0.056
The course of the disease-months	10.83 ± 4.41	10.60 ± 4.30	10.91 ± 4.46	-1.00	0.317
Onset age-years	34.09 ± 12.36	32.81 ± 11.88	34.58 ± 12.51	-2.01	0.045^*^
HAMD	29.43 ± 2.97	28.22 ± 2.60	29.90 ± 2.97	-8.75	< 0.001^*^
HAMA	20.28 ± 3.49	19.59 ± 3.33	20.59 ± 3.52	-3.83	< 0.001^*^
PSS	8.67 ± 4.39	7.81 ± 3.30	9.01 ± 3.70	-4.49	< 0.001^*^
CGI-SI	5.83 ± 0.71	5.58 ± 0.63	5.92 ± 0.72	-6.85	< 0.001^*^
TSH-uIU/mL	3.98 ± 2.47	3.44 ± 1.88	4.19 ± 2.64	-4.92	< 0.001^*^
FT_3_-mmol/L	4.90 ± 0.69	4.84 ± 0.71	4.92 ± 0.69	-1.44	0.151
FT_4_-mmol/L	16.78 ± 3.04	16.81 ± 3.03	16.77 ± 3.03	0.21	0.831
FBG-mmol/L	5.26 ± 0.63	5.18 ± 0.58	5.29 ± 0.65	-2.44	0.015^*^
TC-mmol/L	4.79 ± 0.92	4.73 0.58	4.98 0.96	-11.40	< 0.001^*^
HDL-C-mmol/L	1.31 ± 0.23	1.34 0.21	1.31 0.23	2.20	0.028^*^
LDL-C-mmol/L	2.67 ± 0.74	2.46 0.50	2.75 0.80	-5.50	< 0.001^*^
TG-mmol/L	2.11 ± 1.00	1.23 0.35	2.45 0.97	-20.39	< 0.001^*^
BMI-kg/m^2^	24.18 ± 1.76	24.14 ± 1.66	24.20 ± 1.80	-0.65	0.517
SBP-mmHg	116.3 ± 11.14	114.7 ± 10.22	117.0 ± 11.43	-2.88	0.004^*^
DBP-mmHg	74.62 ± 6.83	73.77 ± 6.04	74.95 ± 7.08	-2.63	0.009^*^
WC-cm	79.78 ± 8.40	80.64 ± 8.47	79.73 ± 8.37	1.51	0.130
Gender				2.42	0.120
Male	333, 33.9%	103, 37.7%	230, 32.5%		
Female	648, 66.1%	170, 62.3%	478, 67.5%		
Education High school and below Bachelor and above	683, 69.6% 298, 30.4%	179, 65.5% 94, 34.4%	504, 69.6% 204, 30.4%	2.94	0.086
Marital status				2.16	0.142
Unmarried	307, 31.3%	95, 34.8%	212, 29.9%		
Married	674, 68.7%	178, 65.2%	496, 70.1%		
Treatment history				1.09	0.296
NO	345, 35.2%	89, 32.6%	256, 36.2%		
YES	636, 64.8%	184, 67.4%	452, 63.8%		
Suicidal history				4.13	0.042^*^
NO	849, 86.5%	246, 90.1%	603, 85.2%		
YES	132, 13.5%	27, 9.9%	105, 14.8%		

HAMD: Hamilton Depression Scale; HAMA: Hamilton Anxiety Scale; PSS: Positive Symptom Subscale; CGI-SI: Clinical Global Impression Scale-Severity of Illness; TSH: thyroid stimulating hormone; FT_3_: free triiodothyronine; FT_4_: free tetraiodothyronine; FBG: fasting blood glucose; TC: total cholesterol; HDL-C: high-density lipoprotein cholesterol; LDL-C: low-density lipoprotein cholesterol; TG: triglycerides; BMI: body mass index; SBP: systolic blood pressure; DBP: diastolic blood pressure; WC: waist circumference. ^*^p<0.05

### Simple correlation analysis of HL and other parameters in patients with MDD

We performed correlation analysis to test the correlation between HL and other variables. We used the chi-square test for the correlation between categorical and categorical variables. We used Spearman correlation analysis for correlation between continuous numerical and categorical variables. Table 2 summarizes the results of the correlation coefficients.

**Table 2:** Simple correlation analysis of HL and other parameters in patients with MDD

Parameters	HL
Age-years	0.057
The course of the disease-months	0.029
Onset age-years	0.061
HAMD	0.269^**^
HAMA	0.131^**^
PSS	0.122^**^
CGI-SI	0.212^**^
TSH-uIU/mL	0.113^**^
FT_3_-mmol/L	0.054
FT_4_-mmol/L	-0.007
FBG-mmol/L	0.063
BMI-kg/m^2^	0.020
SBP-mmHg	0.087^**^
DBP-mmHg	0.064^*^
WC-cm	-0.046
Gender (Male vs. Female)	0.050
Education (High school and below vs. Bachelor and above)	-0.055
Marital status (Unmarried vs. Married)	0.047
Treatment history (No vs. Yes )	-0.033
Suicidal history (No vs. Yes )	0.065^*^

HAMD: Hamilton Depression Scale; HAMA: Hamilton Anxiety Scale; PSS: Positive Symptom Subscale; CGI-SI: Clinical Global Impression Scale-Severity of Illness; TSH: thyroid stimulating hormone; FT_3_: free triiodothyronine; FT_4_: free tetraiodothyronine; FBG: fasting blood glucose; BMI: body mass index; SBP: systolic blood pressure; DBP: diastolic blood pressure; WC: waist circumference. ^*^p < 0.05, ^**^p < 0.01

### Determinants of HL in patients with MDD: based on a binary logistic model

After correlation analysis, we performed a binary logistic regression model (backwards: Wald) with HL as the outcome variable to identify the different independent variables and their relationship with HL in univariate analysis. The results showed that the HAMD score (B = 0.21, *p* < 0.001, OR = 1.23) and CGI-SI score (B = 0.42, *p* = 0.001, OR = 1.52) were risk factors for HL. These results are summarized in Table 3.

**Table 3:** Binary logistic regression analyses of determinants of HL in MDD patients

	Coefficients	Std. error	Wald	*p*	95% CI for EXP (B)		
	B				Exp(B)	Lower	Upper
Constant	-6.43	0.86	53.67				
HAMD	0.21	0.04	31.40	< 0.001^*^	1.23	1.14	1.32
HAMA	-0.51	0.03	3.08	0.080	0.95	0.90	1.00
CGI-SI	0.42	0.13	10.91	0.001^*^	1.52	1.18	1.94

HAMD: Hamilton Depression Scale; HAMA: Hamilton Anxiety Scale; CGI-SI: Clinical Global Impression Scale-Severity of Illness. ^*^p < 0.05

### ROC analysis of influencing factors of HL in MDD patients

**Fig. 1:**
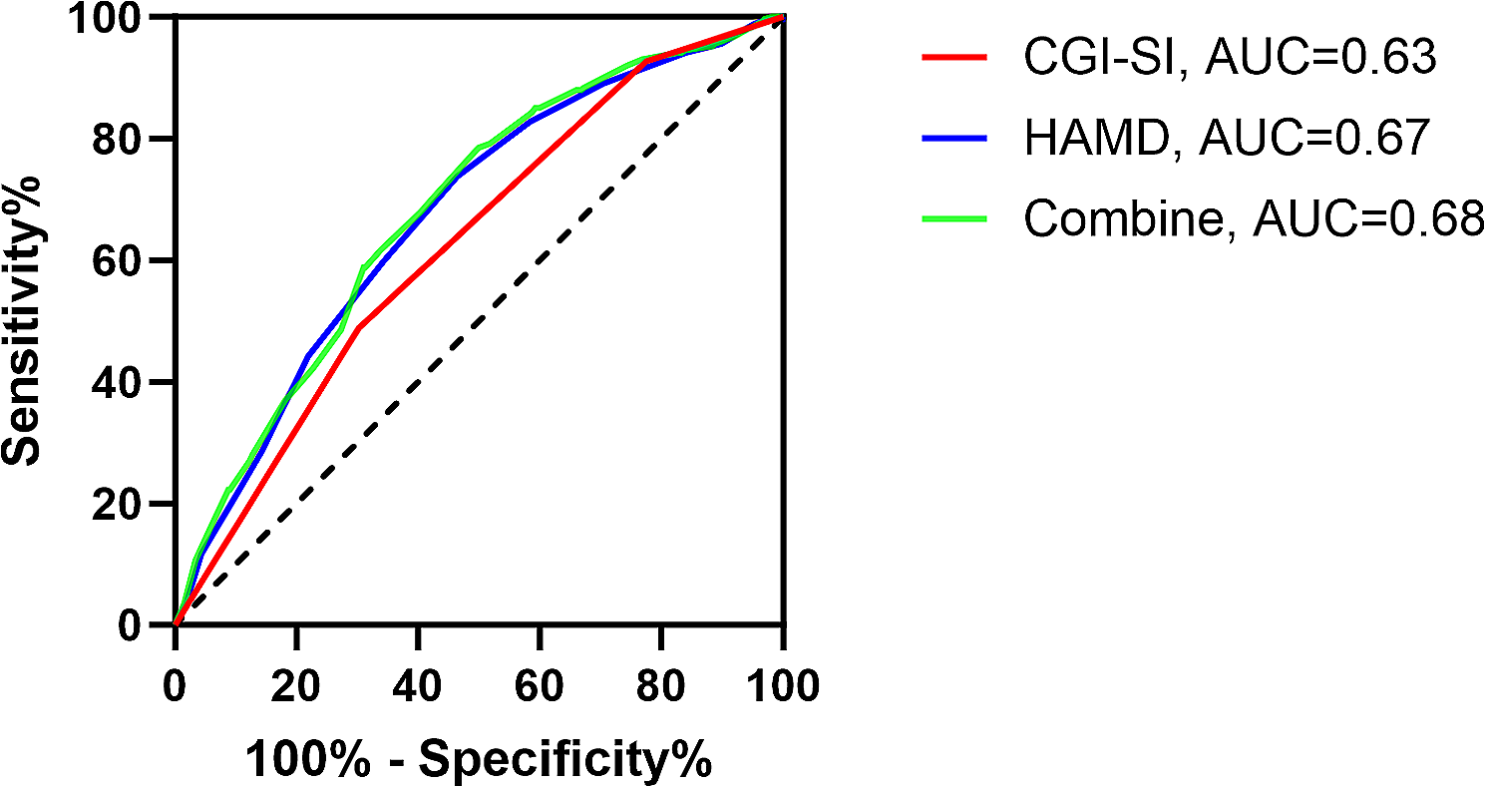
Diagnostic power of CGI-SI and HAMD score for MDD combined with HL: a diagnostic model based on ROC curves. CGI-SI: Clinical Global Impression Scale-Severity of Illness; HAMD: Hamilton Depression Scale

We performed a ROC analysis of the risk factors identified in the binary logistic regression analysis that significantly affected lipid levels in MDD patients (Table 3). The area values under the ROC curve for each risk factor were as follows: the HAMD score was 0.67, and the CGI-SI score was 0.63 (Table 4; Fig. 1). And the combination of these two parameters showed some ability to discriminate HL in MDD patients (AUC = 0.68, *p* < 0.001, 95% CI: 0.64–0.72).

**Table 4:** ROC analysis of causes affecting lipids

	AUC	Cut-off value	Youden index	Sensitivity	Specificity
CGI-SI	0.63	6	0.19	0.70	0.49
HAMD	0.67	30	0.27	0.54	0.74
Combine	0.68		0.28	0.50	0.78

CGI: Clinical Global Impression Scale-Severity of Illness; HAMD: Hamilton Depression Scale. ^*^p < 0.05

Cut-off value rounded up

### Correlation analysis between lipid parameters

We employed Pearson correlation analysis to investigate the relationship between various lipids in MDD patients with combined HL. We discovered a noteworthy and moderately robust (*r* = 0.560) positive correlation between TC and LDL-C at the *p* < 0.01 level. The results are summarized in Table 5.

**Table 5:** Correlation analysis between lipid parameters

	TC	HDL-C	TG	LDL-C
TC	1			
HDL-C	0.158^**^	1		
TG	-0.067	0.016	1	
LDL-C	0.560^**^	0.103^**^	-0.206^**^	1

TC: total cholesterol; HDL-C: high-density lipoprotein cholesterol; TG: triglycerides; LDL-C: low-density lipoprotein cholesterol. ^**^p < 0.01

## Discussion

Our study investigated the reported prevalence of HL and associated factors in patients with MDD who were hospitalized for the first time. The main results of this study were as follows: (1) The prevalence of HL among patients with first-onset MDD was 72.17%, and 708 met the diagnostic criteria for HL. (2) Among patients with MDD who were hospitalized for the first time, there were significant differences between HL and non-HL subgroups in terms of age at onset, history of suicide, some scaled scores associated with clinical symptoms (including HAMA score, HAMD score, PSS score, CGI-SI score) and metabolism-related parameters (including DBP, SBP, FBG, TSH). (3) HAMD score and CGI-SI score were risk factors for the development of HL in patients with MDD who were hospitalized for the first time. (4) We plotted ROC curves. The area under the ROC curve for the CGI-SI and HAMD score and their combined discriminatory ability of the two factors was approximately 63%, 67%, and 68%, respectively. (5) In MDD patients with co-morbid HL, there was a significant moderate-strength positive correlation between TC and LDL-C levels.

First, we reported the prevalence of combined dyslipidemia among first hospitalized MDD patients was 72.17%, with the prevalence of high TC, high LDL-C, low HDL-C, and high TG accounting for 30.17%, 14.68%, 6.52%, and 57.39% of the total number of first hospitalized MDD patients, respectively. We reported a higher prevalence of HL in patients with MDD than in the Chinese population without MDD or any psychiatric disorder [28]. The higher prevalence of HL in MDD patients than in the average population may be related to the use of antidepressants and antipsychotics or poor lifestyle habits, such as low levels of exercise and unhealthy diet, in MDD patients [29, 30]. A national study on the correlates of HL among first-treatment MDD patients showed that the proportion of MDD patients with co-morbid HL was 61% (1048/1718) [31], which is lower than the 72.17% we reported. However, more than half of the population in our study (64.8%) had a history of outpatient treatment, and the use of medication may explain the different prevalence of HL we reported [32]. Our definition of dyslipidemia was also more stringent, so more patients were included in the non-HL subgroup.

Regarding foreign studies, to our knowledge, current data reporting the prevalence of HL in the MDD population are incomplete. A retrospective study from the United Kingdom reported a prevalence of 41.6% for high TG and 34.2% for low HDL-C in depressed patients [33], and the prevalence of high TG was generally consistent with that reported in the German study (43.1%) [34]. Although there is heterogeneity in the current relevant studies, the reasons for these heterogeneities may be related to demographics, age, and sociocultural characteristics. However, they largely support our finding that the prevalence of HL is higher in patients with MDD.

Second, our study showed significant differences between the HL subgroup and the non-HL subgroup on several clinical symptom scales (HAMD, HAMA, CGI-SI, PSS), metabolism-related indicators (FBG, SBP, DBP, TSH), presence of a history of suicide, and age at onset. Existing studies have shown that patients with combined lipid metabolism have higher HAMD scores, HAMA scores, PSS scores, and TSH levels than patients with MDD without combined abnormal lipid metabolism [35]. Patients with MDD with or without HL also exhibit variability in metabolic indicators. Previous studies have shown a biphasic relationship between depression and metabolism, with an increased risk of HL, hypertension, and hyperglycemia in MDD patients [36, 37]. It may be associated with increased central and peripheral activation of the immunometabolic or endocrine system [38, 39]. A study of lipid disorders and suicide risk showed an increased risk of suicide attempts in MDD patients with HL [40]. The biological mechanisms underlying the association between lipid profile and suicide in MDD patients remain unclear; the possible influence of lipid profile on the 5-hydroxytryptamine system may play an important role [41]. Studies on the effect of age at onset on lipids in MDD patients are scarce, and in available research, we found that depressed patients with childhood onset showed higher TG levels and lower HDL-C levels [42].

Third, we reported two risk factors: the HAMD and CGI-SI score, which implied that HL was more likely to occur in MDD patients with severe clinical symptoms, consistent with previous studies [43, 44]. However, the relationship between depression and metabolism may be bidirectional, as a longitudinal study in Finland found that a pattern of sharply elevated triglyceride levels throughout childhood and early adulthood may be associated with an increased risk of depressive symptoms in adulthood [45]. Furthermore, numerous studies support that more disturbed metabolic indicators appear to be associated with depression severity in depressed patients [16, 46, 47]. One study found that TNF-alpha and cytosolic interleukin (I)-6 transcriptional signaling may be increased in patients with refractory depression, implying that lipid peroxidation may inhibit therapeutic response in depressed patients [48]. Interestingly, in another study, infliximab improved treatment response and HL in patients with refractory depression by inhibiting TNE signaling, and the reduction of inflammation has a positive role in driving both lipid metabolism and clinical symptomatic improvement in depressed patients [49], i.e., inflammation may be a common pathomechanism for both.

In addition, we plotted ROC curves. The area under the ROC curve for the CGI-SI and HAMD score and the combined discriminatory power of these factors were approximately 63%, 67%, and 68%, respectively, which implies that the severity of the patient’s condition and the severity of the depressive symptoms have some diagnostic power for the presence of HL in patients with MDD.

Finally, we investigated the relationship between different lipid parameters in MDD patients with HL. We found a significant and moderately strong positive correlation between TC and LDL-C. Considering that LDL-C is a part of TC, we speculated that LDL-C may more closely influence elevated TC in patients with MDD. Between the other types of lipid parameters, we did not find significant correlations or the correlations were so weak that they might not be of practical significance.

There are several limitations to our study. First, as a cross-sectional study, we could not determine the causal relationship between abnormal lipid metabolism and various factors. Second, although our sample included only Chinese people, our findings may not be generalizable and may not apply to populations in other countries due to differences in sociodemographic characteristics. Third, our sample consisted mainly of patients with an urgent need for hospitalization, usually in the acute phase of the disease. Therefore, our findings do not apply to patients with stable MDD. In addition, we could not exclude the effects of confounding factors, such as the effects of patients’ diet, alcohol consumption, and physical activity on lipids. The use of antidepressants also added to the study’s confounding factors, considering that 64.8% of our sample had a history of outpatient treatment. Finally, to minimize the impact of the intervention effect on the study results and more accurately assess the impact of other potential factors on lipids in patients with MDD, we chose to exclude patients who had received lipid-lowering therapy. However, this may also have led to an underestimation of the prevalence of co-morbid HL in MDD in our study. To remedy the shortcomings of the current study, we plan to conduct a more in-depth prospective study in future studies.

In conclusion, this study demonstrated a high prevalence of HL in first-time hospitalized MDD patients. Higher HAMD and CGI-SI scores were risk factors for the development of HL in MDD patients, and HAMD and CGI-SI scores predicted the severity of HL. These results suggest that timely improvement of the clinical status of at-risk individuals may help reduce their risk of developing dyslipidemia. It also implies that the presence of HL may indicate more severe depressive symptoms in patients with MDD. However, due to individual differences (including other health problems, lifestyle, genetic risk, etc.), the use of a fixed threshold to define HL may not accurately reflect the actual risk profile of patients with MDD. Therefore, clinicians need to evaluate patients carefully on a case-by-case basis. In addition, clinicians should pay close attention to lipid metabolism in patients with major depression. Given the interaction between depressive symptoms and HL, lipid-lowering therapy should be initiated immediately in patients with significant depression who meet the diagnostic criteria for HL.

## Data Availability

The datasets used and/or analyzed during the current study are available from the corresponding author upon reasonable request.

## Abbreviations

HL: hyperlipidemia
MDD: Major Depressive Disorder
OS: Oxidative Stress
ROS: Reactive Oxygen Species
TNF: Tumor Necrosis Factor
HAMD: Hamilton Depression Scale
HAMA: Hamilton Anxiety Scale
PSS: Positive Symptom Subscale
CGI-SI: Clinical Global Impression Scale-Severity of Illness
TSH: Thyroid Stimulating Hormone
FT_3_: Free Triiodothyronine
FT_4_: Free Tetraiodothyronine
FBG: Fasting Blood Glucose
TC: Total Cholesterol
HDL-C: High-Density Lipoprotein Cholesterol
LDL-C: Low-Density Lipoprotein Cholesterol
TG: Triglycerides
BMI: Body Mass Index
SBP: Systolic Blood Pressure
DBP: Diastolic Blood Pressure
WC: Waist Circumference
